# The transcriptional regulator gene E2 of the Human Papillomavirus (HPV) 16 influences the radiosensitivity of cervical keratinocytes

**DOI:** 10.1186/1748-717X-7-187

**Published:** 2012-11-07

**Authors:** Katja Lindel, Stefan Rieken, Sigrid Daffinger, Klaus J Weber, Ethel-Michele de Villiers, Jürgen Debus

**Affiliations:** 1 Dept. of Radiation Oncology and Radiotherapy, University of Heidelberg, Im Neuenheimer Feld 400, Heidelberg 69120, Germany; 2 Division of Tumorvirus Characterisation, Deutsches Krebsforschungszentrum, Im Neuenheimer Feld 242, Heidelberg, 69120, Germany

**Keywords:** Human Papillomavirus, Radiosensitvity, E2-gene, Cervical keratinocytes

## Abstract

**Background:**

Clinical studies have demonstrated that HPV induced tumors constitute a specific subclass of cancer with a better response to radiation treatment. The purpose of this study was to investigate meaning of viral E2-gene for radiosensitivity.

**Methods:**

W12 cells contain episomal HPV 16 genomes, whereas S12 cells, which derive from the W12 line, contain HPV DNA as integrated copies. Clonogenic survival was analyzed using 96-well *in vitro* test. Using flow cytometry cell cycle analyses were performed. Expression of *pRb* and *p53* were analyzed using intracellular staining.

**Results:**

W12 cells (intact E2 gene) showed a lower survival fraction than S12 cells. W12 cells developed a G2/M block 24 h after irradiation with 2 Gy whereas S12 showed no G2/M bloc. After irradiation S12 cells developed polyploidy and *pRb*-positive cells decreased. W12 cells showed no change of *pRb*-positive cells.

**Conclusions:**

Depending on E2 gene status differences in cell cycle regulation might cause radioresistance. The E2/E7/*pRb* pathway seems to influence HPV-induced radiosensitivity. Our experiments demonstrated an effect of HPV on radiosensitivity of cervical keratinocytes via viral transcription regulator E2 pathway.

## Background

Cervical cancer is considered to be a sexually transmitted disease and has been correlated with Human Papillomavirus infection (HPV) [1]. Besides a number of prognostic factors like depth of stromal invasion, tumor differentiation or nodal involvement, presence of Human Papillomavirus has been suggested an important marker of disease severity in cervical cancer [2]. The viral genome is organized into three general segments of unequal size: long control region (LCR), early (E) and late (L) genes. Acting as transcriptional activator or repressor, E2 protein regulates virus transcription and genome replication [3]. Loss of E2 gene integrity seems to play a role for outcome and local control in cervical carcinomas [4,5]. It controls transcription of oncogenes E6 and E7 which manipulate cell cycle and ability of apoptosis [6]. There may be a possible correlation between radiotherapy, E2 function and outcome [4,7,8]. The E6 oncoprotein can form a complex with host cell *p53* tumor suppressor protein and inducing *p53* degradation and overcoming G_1_/S checkpoint control in DNA-damaged cells [9]. E7 oncoprotein binds to hypophosphorylated *pRb* form resulting in its degradation and inappropriate release of E2F transcription factor [10]. Pre-clinical data arising from comparison between non-HPV-tumor cells and their counterparts transfected with sequences of HPV genome should be interpreted with caution because artificial induced expression might not mirror *in vivo* reality. To avoid artificial uncertainties we used W12/S12 cell model derived from a low grade cervical lesion by Stanley MA et al. 1989 [11] to evaluate the influence of E2 on intrinsic radiosensitivity of cervical cells to support the hypothesis of E2-gene status as a predictive marker for therapeutic outcome in cervical cancer patients.

## Methods

### Cell lines and cell culture

W12 cell line was derived from a low grade cervical lesion by Stanley MA et al. 1989, and is unique among HPV16-containing cell lines in carrying its HPV 16 genome as a multicopy episome [11]. We made use of a pair of isogenic cell lines, W12 and S12 to compare difference of survival after irradiation. W12 cells contain episomal HPV 16 genomes, whereas S12 cells, which derived from the W12 line, contain HPV DNA as integrated copies [12].

W12 cells were cultured with lethally irradiated Swiss 3T3 feeder cells and in medium consisting a mix of one-quarter Dulbecco’s modified eagle’s medium (Gibco) and three-quarters Ham F-12 medium (Gibco) containing 5% fetal calf serum, penicillin, streptomycin, an supplements (all from Sigma) as follows: 8.4.ng of cholera toxin/ml, 5 μg of insulin/ml, 24.3μg of adenine/ml, 0.5 μg of hydrocortisone/ml and 10 ng of epithelial growth factor per ml. Cells were split when reached 80% confluence. S12 cells were obtained by collecting surviving W12 cells cultured without feeder layer support.

### E2-gene specific PCR

HPV16-positive cells were tested for an intact E2 gene in three separate amplification reactions which allows amplifying three amplicons of different length determining integrity of E2-gene [8]. The procedure and primer sequences used were as described in the same reference.

### Clonogenic growth assay and irradiation

Clonogenic survival was analyzed by using 96-well *in vitro* test as followed: 1-100 cells per well were seed. The plates were examined with an inverted phase contrast microscope at intervals of 7, 10, 14 days. A well was considered positive when a colony in it reached a size of 50 cells or more. Cells were fixed with 70% for ten minutes prior staining with 0.1% methylene–blue. After staining weels were washed with destilled water. Plating efficiency (PE) was calculated using poisson statistics according to formula PE = -ln (neg wells/total wells)/ number of cells plated per well [13]. In radiation experiments fraction of survival was determined by dividing number of positive wells/plate/number of cells plated per well in irradiation group by number of positive wells/plate/number of cells plated per well in control plates. At least three plates were used for each group.

Cells were irradiated with singles doses of 0 Gy, 1 Gy, 2 Gy, 3 Gy, 4 Gy, 5 Gy and 7 Gy. In such experiments, an increasing number of cells plated for each increment in radiation dose. Therefore, effect of cell number per well on plating efficiency was evaluated. Plating densities of 1-10 cell/weel were tested. Although number of wells with colonies increased with higher cell density, plating efficiency was not effected by number of cells. When 10 cells/well were used all wells in this set of experiments contained colonies.

Survival curves were based on number of positive wells or colonies in each irradiated group as a fraction of that in control group. Survival curves where calculated using Sigma Plot 8.0. At least three experiments where performed for each dose point.

### Cell cycle analyses

Cell cycle analyses were performed after 0 h, 6 h, 12 h, 24 h, 48 h and 7 days irradiation with 2 Gy and 7 Gy using flow cytometry using Propidium-Iodid (PI)-staining as described elsewhere [14]. Data were collected by using FACScan flow cytometry, and results were analyzed by using cellquest software (both from Becton Dickenson). For each sample, 10000 events were collected, and aggregated cells were gated out.

### Intracellular cytokine staining: pRb and p53

The retinoblastoma gene encodes a nuclear phosphoprotein which is expressed in most normal cells and acts as a tumor suppressor. An underphophorylated form of Rb binds to viral oncogene HPV-E7 [3]. Clone G3-245 recognizes an epitope between amino acids 300-380 of the human retinoblastoma protein (pp110-114 Rb).

Wildtype *p53* formes specific complexes with several viral oncogenes including HPV-E6 and plays a role as checkpoint protein for DNA damage during G1/S-phase of cell cycle [9]. Clone G59-12 recognize mutant and wild type human, mouse and rat p53 suppressor protein.

The G3-245 or G59-12 and MOPC-21 FITC (a mouse IgG1 isotype control) conjugates are matched and F/P ratios determined experimentally by flow cytometric analysis.

Details of the procedure are described as follows:

Ethanol fixated cells were washed two times in cold PBS then resuspended in Fixation/Permialisation solution Perm/Wash^TM^ BD (1x10^6^ cell/ml) for 30 min at 4°C and pelleted by centrifugation. Afterwards buffer was removed and cells were washed two times in fresh Perm/Wash^TM^ BD buffer. Thoroughly resuspended cells were subjected to intracellular cytokine staining by incubating in 100μl Perm/Wash^TM^ BD buffer containing 20μl of Fluorochrome*-*conjugated antibody Rb-ak (FITC Mouse Anti-Human Retinoblastoma Anti-Body from Becton Dickinson, BD-Set:# 556538 Clone G3-245) for 24h at 4°C temperature in the dark. After washing with Perm/Wash^TM^ BD cells were pelleted and resuspended cells in 0,5 ml Perm/Wash^TM^ BD for flow cytometric analysis. The same procedure was performed for p53 staining using 20μl of Fluorochrome*-*conjugated antibody p53-ak (FITC Mouse Anti-Human p53 Anti-Body from Becton Dickinson, Clone G59-12).

### Flow cytometric analysis

Stained cells were analyzed using FACSCan flow cytometry (BD) equipped with a air-cooled 488 nm argon-ion laser. Data acquisition and analysis were performed using FACSComp and CELLQuest (version 3.4) software. A total event of 10 000 cells were acquired for each sample. Data were expressed as geometric mean fluorescence intensity and as ratio between fluorescence emission of sample cells and that of isotypic control (P/N ratio; positive/negative). In each case negative control were cells treated as described above without Rb-ak staining or p53-ak staining. Isotypic control were cells treated with isotype–matched control of irrelevant specificity from FITC Mouse IgG1 Isotype control (BD-Set# 556538) instead of Rb-ak staining or p53-ak staining. Analyses were performed after 0 h and 24 h irradiation with 2 Gy and 7 Gy.

## Results

### Intact E2-gene leads to higher radiation sensitivity in cervical cells

**Figure 1:**
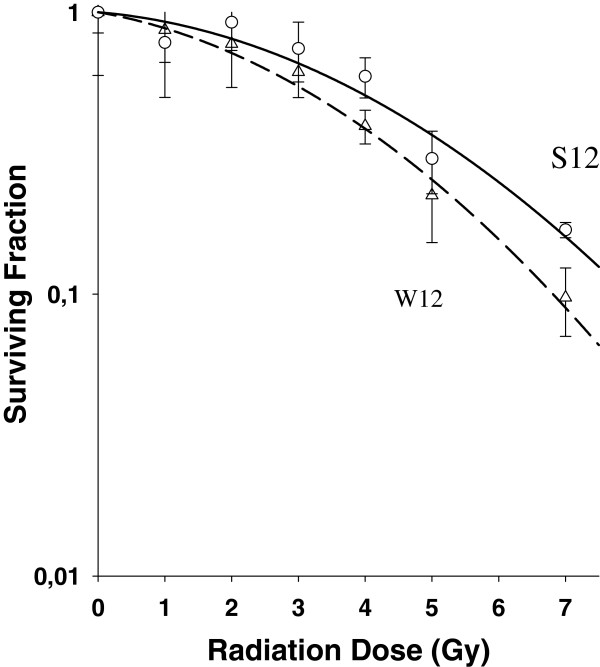
**Survival curves (Sigma Plot 8.0) of W12 cells (intact E2-gene) compared to S12 cells (disrupted E2-gene).** Legend 1: Clonogenic survival was analyzed by using 96-well in vitro test. The E2 gene of S12 cells (passage 88 – 103) was disrupted in the E2C region. W12 cells (passage 8 –14) with an intact E2 gene showed a higher radiosensitivity with a radiation enhancement factor of 1.5 (4 Gy).

The E2 gene of S12 cells (passage 88 – 103) was disrupted in the E2C region. W12 cells (passage 8 –14) with an intact E2 gene showed a higher radiosensitivity with a radiation enhancement factor of 1.5 (4 Gy) (Figure 1).

### E2-gene alters cell cycle regulation after radiation

**Figure 2:**
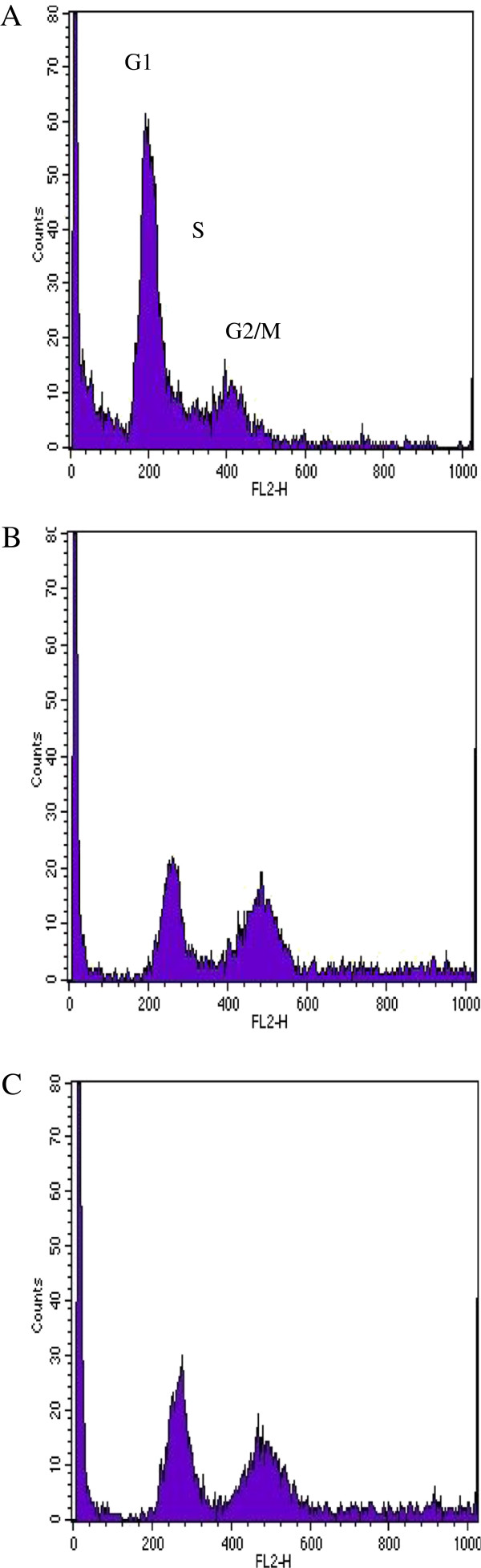
**Plot of flow cytometry using PI-staining for cell cycle analysis of W12 cells (intact E2-gene).** Legend 2: **A** = control group, **B** = 2 Gy (24 h), **C** = 7 Gy (24 h): Figure 2 shows flow cytometric analysis of W12 cells 24 h after irradiation with 0 Gy, 2 Gy and 7 Gy. In control group 64% of cells were in G1, 9% in S-phase and 28% in G2/M-phase. After irradiation with 2 Gy the amount of cells in G2/M-phase increased to 43% and after irradiation with 7 Gy to 52%. In G1-phase were 49% and 40% , in S-phase 8% and 7% after irradiation with 2 Gy and 7 Gy, respectively.

Compared to S12 cells, W12 cells (intact E2-gene) showed a G2/M-block 6 h-24 h after irradiation with 2 Gy. 48 h after 2 Gy irradiation W12 cells entered cell cycle again. After irradiation with 7 Gy G2/M-block of W12 cells lasted at least 72 h and resolved after 7 days. Figure 2 shows flow cytometric analysis of W12 cells 24 h after irradiation with 0 Gy, 2 Gy and 7 Gy. In control group 64% of cells were in G1, 9% in S-phase and 28% in G2/M-phase. After irradiation with 2 Gy the amount of cells in G2/M-phase increased to 43% and after irradiation with 7 Gy to 52%. In G1-phase were 49% and 40% , in S-phase 8% and 7% after irradiation with 2 Gy and 7 Gy, respectively.

**Figure 3:**
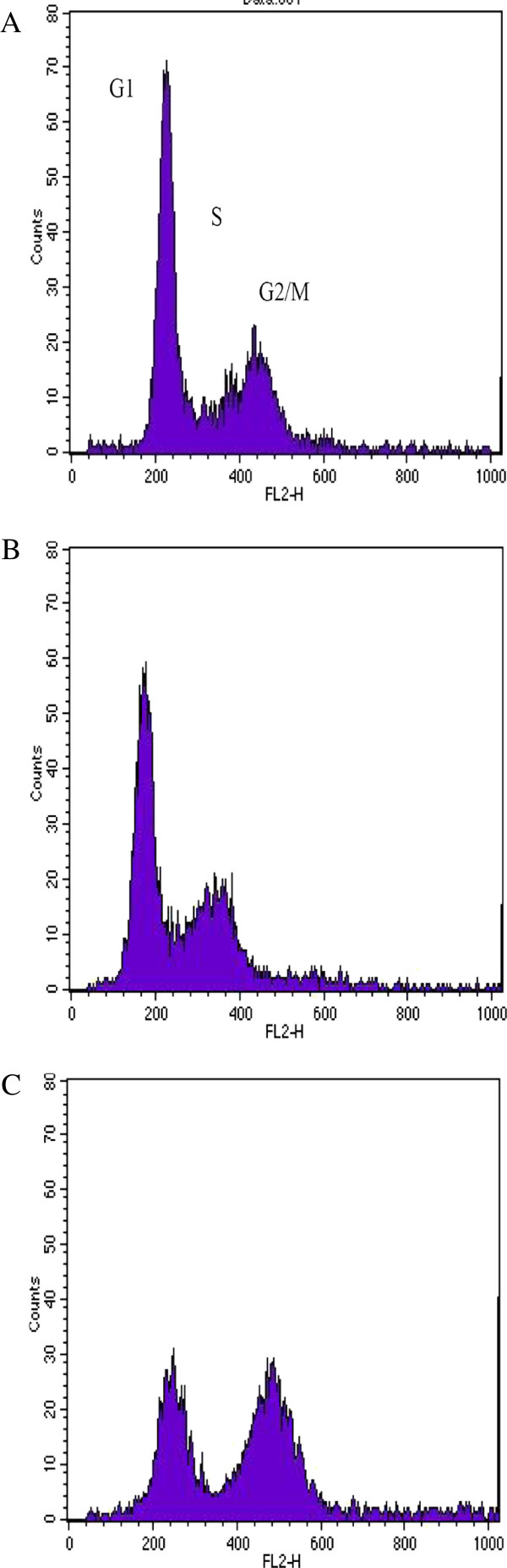
**Plot of flow cytometry using PI-staining for cell cycle analysis of S12 cells (disrupted E2-gene).** Legend 3: **A** = control group, **B** = 2 Gy (24 h), **C** = 7 Gy (24 h): Figure 3 shows flow cytomtric analysis of S12 cells 24 h after irradiation with 0 Gy, 2 Gy and 7 Gy. In the control group 58% of cells were in G1, 14% in S-phase and 27% in G2/M-phase. After irradiation with 2 Gy cell distribution remained the same. After irradiation with 7 Gy 50% of cells were in G2/M-phase, 41% in G1-phase and 9% in S-phase.

S12 cells (disrupted E2-gene) showed no effect like G2/M- or G1-block after irradiation with 2 Gy. S12 cells showed a G2/M-block 12 h to 48 h after irradiation with 7 Gy. 72 h after treatment S12 cell entered cell cycle again. Figure 3 shows flow cytomtric analysis of S12 cells 24 h after irradiation with 0 Gy, 2 Gy and 7 Gy. In the control group 58% of cells were in G1, 14% in S-phase and 27% in G2/M-phase. After irradiation with 2 Gy cell distribution remained the same. After irradiation with 7 Gy 50% of cells were in G2/M-phase, 41% in G1-phase and 9% in S-phase.

**Figure 4:**
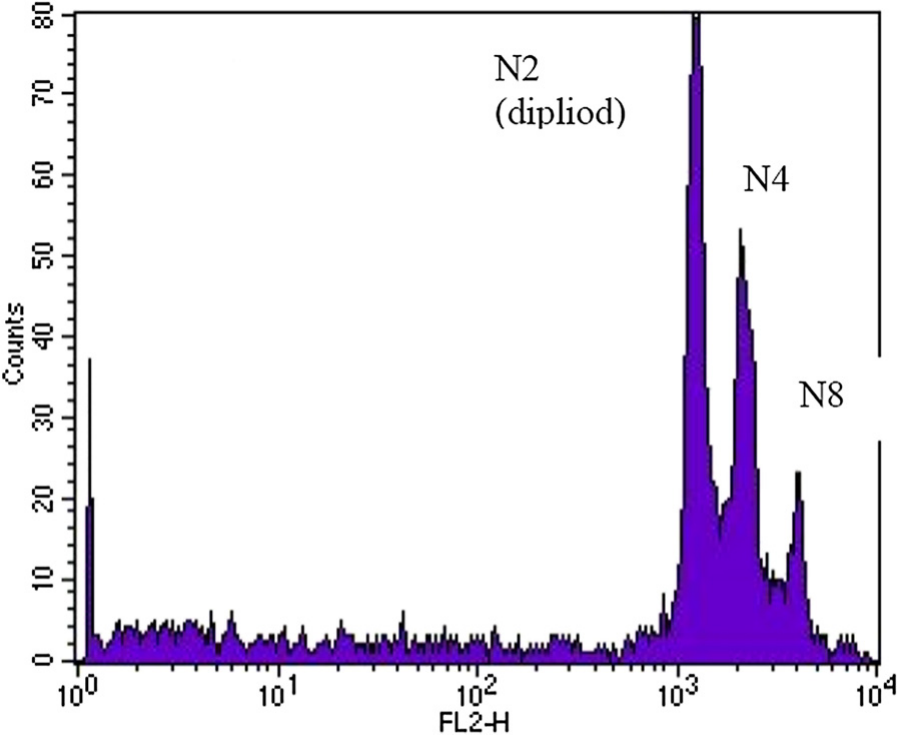
Plot of flow cytometry using PI-staining: S12 cells (disrupted E2-gene) developed aneuploidy 48 h after irradiation with 7 Gy.

S12 cells developed aneuploidy 48 h after 7 Gy irradiation (Figure 4). Both cell lines did not develop a G1-block (Figure 2 and 3).

### E2-status changes pRb-expression after irradiation, but p53-expression is not altered

**Figure 5:**
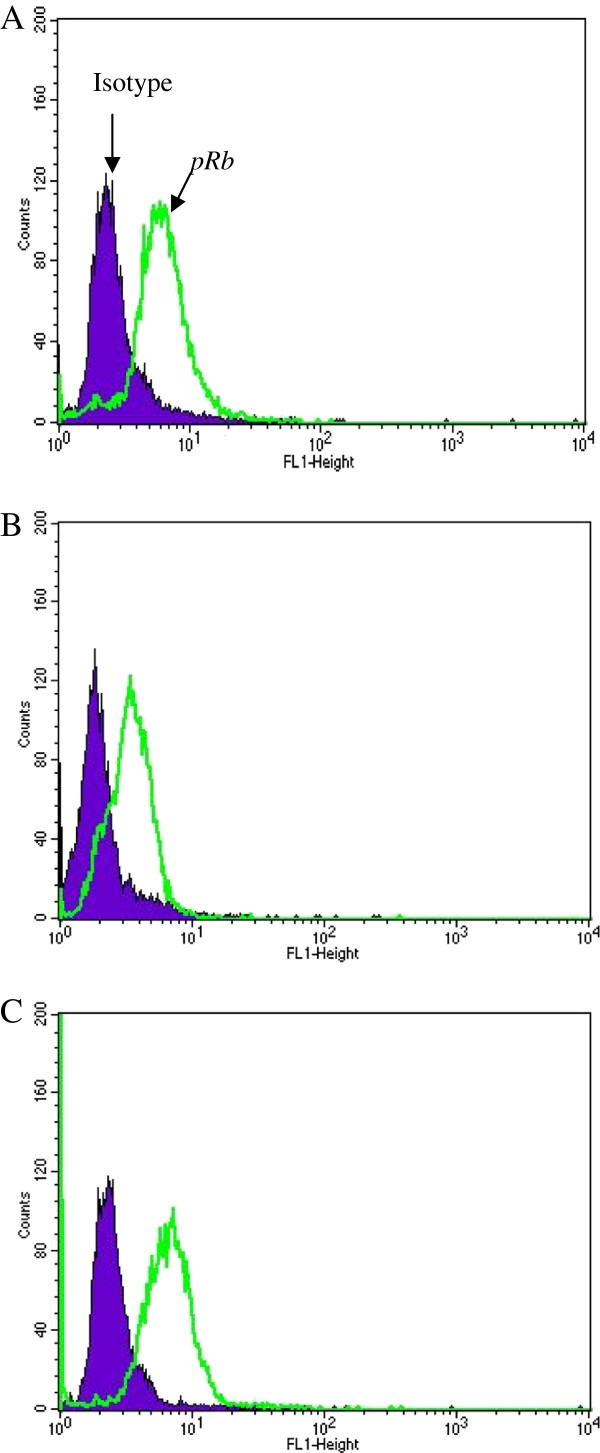
**Plot of flow cytometry using intracellular staining: No change of *pRB *labeling in W12 cells (intact E2-gene) after irradiation.** Legend 5: **A** = control group, **B** = 2 Gy, **C** = 7 Gy: In each case negative control were cells treated as described above without Rb-ak staining. Isotypic control were cells treated with isotype–matched control of irrelevant specificity from FITC Mouse IgG1 Isotype control (BD-Set# 556538) instead of Rb-ak staining or p53-ak staining. Analyses were performed after 0 h and 24 h irradiation with 2 Gy and 7 Gy.W12 cells (intact E2-gene) showed 80%, 77% and 80% *pRb*-labeled cells after irradiation with 0 Gy, 2 Gy and 7 Gy, respectively.

81% of S12 cells (disrupted E2-gene) were positive for *pRb*-expression in the control group. 24 h after irradiation with 2 Gy and 7 Gy *pRb*- labeled cells dropped down to 2.3% and to 5%, respectively (Figure 5).

**Figure 6:**
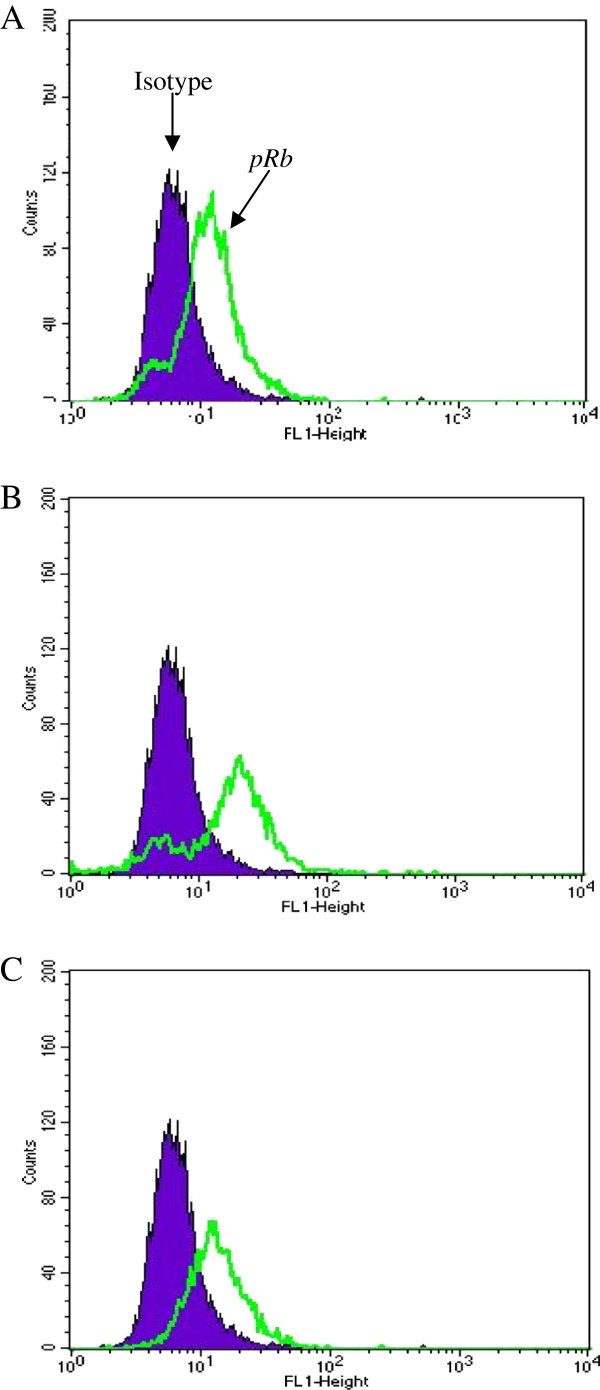
**Plot of flow cytometry using intracellular staining: Change of *pRB *labeling in S12 cells (disrupted E2-gene) after irradiation.** Legend 6: **A** = control group, **B** = 2 Gy, **C** = 7 Gy: 81% of S12 cells (disrupted E2-gene) were positive for *pRb*-expression in the control group. 24 h after irradiation with 2 Gy and 7 Gy *pRb*-labeled cells dropped down to 2.3% and to 5%, respectively.

W12 cells (intact E2-gene) showed 80%, 77% and 80% *pRb*-labeled cells after irradiation with 0 Gy, 2 Gy and 7 Gy, respectively (Figure 6).

There was no significant overexpression of *p53* in both cell lines and no change after irradiation with 2 Gy or 7 Gy.

## Discussion

Reasons for an increased radiocurability of HPV-positive tumors are not defined yet. Disruption of the viral E2-gene has been shown to be associated with poor outcome in patients with cervical cancer [4,7,8]. Not only in cervical cancer, but also in HPV-positive head and neck cancer E2-protein may be relevant for treatment success [15]. There is still a debate about direct influence of HPV on radiosensitivity. Because for these clinical reports and the referred interference of E2-protein with regulation of apoptosis and cell cycle control [6,16] we established the W12/S12 cell system to analyze influence of E2-gene status on radiosensitivity. This cell model mimics the natural way of integration of virus leading to disruption of the E2-gene [11,12]. We could demonstrate that a disrupted E2-gene leads to radioresistance, because W12/S12 cells differ only in E2-gene status, molecular tumor background is identical. Inactivation of normal function of tumor suppressor proteins *pRb* and *p53* are important steps in human cervical carcinogenesis. Mutations of tumor suppressor genes are rare in HPV-positive cervical cancer cells [17]. The loss of the E2-gene causes reduced proapoptotic signals, as well as deregulation of expression of the oncogenes E6 and E7 [18]. In response to DNA-damaging, *p53*-levels increase by a posttranscriptional mechanism resulting in arrest via inhibition of cyclin-associated kinase activity at G1/S interface of cell cycle [19]. Loss of *p53* or expression of mutant *p53* results in failure to arrest G1 [20,21]. E6 facilitates degradation of p53 through its association with an accessory protein E6-AP, a component of ubiquitin proteolytic pathway [17,22,23]. In both cells types we could not detect a G1-arrest, furthermore there was no significant expression of *p53* in both cell types detectable. HPV E6 seems to inhibit *p53* effectively independently of expression of E2. Pang et al. investigated the effect of E6 and its isoforms on radiosensitivity by transfecting oral squamous cell carcinoma cells with *p53* mutations. They found a radiosensitizing effect induced by E6. E6 also induced a G1-cells loss and increased apoptosis [24]. We could not demonstrate these effects in both cells types. Liu et al. found also a radiosensitizing effect of E6 [25], but others have reported opposite effect or no effect in keratinocytes and other cell types exposed to ionizing radiation [26-29].

We found a difference in cell cycle regulation regarding G2/M checkpoint. Cells with an intact E2-gene showed a G2/M-block after irradiation with 2 Gy, but cells with a disrupted E2-gene needed higher doses to induce a G2/M-block. This suggests that in cells with an intact E2-gene expression of functional *p53* after DNA-damage is dose dependent, because *p53* is required for sustaining G2/M-arrest [30]. Data showed that a given dose of radiation induces a longer G2/M-delay in radiosensitive cell lines than in matched normal or resistant cells [31]. This studies confirm our findings of S12 cells entering cell cycle after 72 h, whereas W12 cells remained in G2/M-block more than 72 h. Furthermore cells with a disrupted E2-gene developed aneuploidy after irradiation but cells with an intact E2-gene did not. Liu et al. demonstrated that E6 is capable of inducing DNA replication after postmitotic checkpoint arrest and induces polyploidy independent of *p53*[32]. P53 plays a key role in mediating postmitotic checkpoint and because E6 targets *p53* for degradation, E6 induces polyploidy through inactivation of *p53*[33,34]. Because we could not find aneuploidy in cells with intact E2-gene our experiments indicate that E2-status influences effect of E6 on inducing aneuploidy and is triggered by irradiation. Experiments by Liu et al. showed amount of cells undergoing apoptosis was inversely correlated with polyploidy, suggesting that polyploid cells were subjected to undergo apoptotic elimination and E6 can inhibit polyploidy-associated apoptosis [32]. Radioresistence may be related to aneuploidy and inhibition of apoptosis by E6 in E2-disrupted cells.

We found down-regulation of *pRb* in cells with disrupted E2-gene after irradiation with 2 and 7 Gy. As previously shown, E7 oncoprotein binds to hypophosphorylated *pRb* form resulting in its degradation and inappropriate release of E2F transcription factor [10]. Decrease of *pRb* is therefore an indirect sign of an increased expression of E7 after irradiation dependent on E2-gene status. Santin et al. have reported that high doses of IR (12.5-100 Gy) could increase E6/E7 expression in cervical carcinoma cell lines [35]. Our experiments indicate that disrupted E2-gene in HPV positive cells lead to an over-expression of the oncogene E7 with a down-regulation of pRb. Abdulkarim et al. could demonstrate that restoration of *pRb* with cidofivor leads to increased radiosensitivity [36]. DeWeese et al. found low dose rate radiotherapy led to both G1 and G2 arrest expressing E6, but in cells expressing both E6 and E7, cells arrested only in G2. Despite this difference cell cycle arrest, no difference in clonogenic survival was seen [28]. Gammoh et al. could demonstrate that activity of E7 can be controlled through a direct interaction with E2, resulting in an inhibition of the activity of E7 [37] and that E7-induced degradation of *pRb* was rescued [38].

Our experiments confirm lack of G1-arrest independent of E2-gene status. The up-regulation of E7 reducing functional *pRb* seems to be an essential factor for enhanced radioresistance of cells with a disrupted E2-gene.

## Conclusion

Our experiment support the hypothesis that better prognosis of patients with E2-gene positive cervical cancer is determined by a better response to radiotherapy compared to HPV-positive cancers without an intact E2-gene. Not only differences in cell cycle regulation, but also regulation of expression of E7 might contribute to E2-dependent radioresponse.

## Competing interest

The authors declare that they have no competing interests.

## Authors’ contributions

KL conceived of the study design, performed all experiments and wrote the manuscript. SD carried out irradiation of the cells and FACS analysis. SR and KJW helped to analyze experiments. EMD contributed with regard to content and scientific context. JD conceived of the study and helped to write and finalize the manuscript. All authors read and approved the final manuscript.
